# Degeneration of Melanin-Containing Structures Observed Longitudinally in the Eyes of SOD1^−/−^ Mice Using Intensity, Polarization, and Spectroscopic OCT

**DOI:** 10.1167/tvst.11.10.28

**Published:** 2022-10-19

**Authors:** Conrad W. Merkle, Marco Augustin, Danielle J. Harper, Martin Glösmann, Bernhard Baumann

**Affiliations:** 1 Center for Medical Physics and Biomedical Engineering, Medical University of Vienna, Austria; 2 Core Facility for Research and Technology, University of Veterinary Medicine Vienna, Austria

**Keywords:** optical coherence tomography, melanin, SOD1 knockout mice, spectroscopy, age-related macular degeneration

## Abstract

**Purpose:**

Melanin plays an important function in maintaining eye health, however there are few metrics that can be used to study retinal melanin content in vivo.

**Methods:**

The slope of the spectral coefficient of variation (SSCoV) is a novel biomarker that measures chromophore concentration by analyzing the local divergence of spectral intensities using optical coherence tomography (OCT). This metric was validated in a phantom and applied in a longitudinal study of superoxide dismutase 1 knockout (SOD1^−/−^) mice, a model for wet and dry age-related macular degeneration. We also examined a new feature of interest in standard OCT image data, the ratio of maximum intensity in the retinal pigment epithelium to that of the choroid (RC ratio). These new biomarkers were supported by polarization-sensitive OCT and histological analysis.

**Results:**

SSCoV correlated well with depolarization metrics both in phantom and in vivo with both metrics decreasing more rapidly in SOD1^−/−^ mice with age (*P* < 0.05). This finding is correlated with reduced melanin pigmentation in the choroid over time. The RC ratio clearly differentiated the SOD1^−/−^ and control groups (*P* < 0.0005) irrespective of time and may indicate lower retinal pigment epithelium melanin in the SOD1^−/−^ mice. Histological analysis showed decreased melanin content and potential differences in melanin granule shape in SOD1^−/−^ mice.

**Conclusions:**

SSCoV and RC ratio biomarkers provided insights into the changes of retinal melanin in the SOD1^−/−^ model longitudinally and noninvasively.

**Translational Relevance:**

These biomarkers were designed with the potential for rapid adoption by existing clinical OCT systems without requiring new hardware.

## Introduction

Age-related macular degeneration (AMD) is a leading cause of severe vision loss, particularly in developed countries, and is predicted to affect around 300 million people worldwide by 2040.^1^ AMD can be classified as wet or dry, with dry AMD accounting for 85% to 90% of AMD cases.^2^ Late-stage dry AMD is characterized by geographic atrophy, which causes progressive degeneration of macular photoreceptors. Geographic atrophy in turn has been linked to choroidal atrophy^3^; however, the exact nature of the relationship remains unclear. One proposed mechanism for photoreceptor cell loss in AMD relates to the age-dependent decrease of melanin in the retinal pigment epithelium (RPE), which has been observed in humans.^4^ Melanin plays a photoprotective role in the retina,^5^ scavenging free radicals^6^ and reactive oxygen species,^7^ thereby reducing oxidative stress caused by higher energy visible light.^8^ As such, retinal melanin content could prove to be a useful biomarker for understanding and tracking the development of retinal diseases such as AMD.

Despite the potential importance of imaging melanin in the eye, there are few in vivo techniques for doing so. Much of the previous literature on melanin content in the human eye relies on ex vivo tissue analysis that is often destructive to the tissue sampled.^4^^,^^9^ Methods such as fundus photography,^10^ fundus reflectometry,^11^^,^^12^ and near-infrared autofluorescence imaging^13^ have been used to provide two-dimensional enface maps of melanin content in the retina in vivo but lack the axial resolution to easily separate RPE and choroidal melanin without complex models. Other methods such as polarization-sensitive optical coherence tomography (PS-OCT)^14^^–^^16^ and photoacoustic imaging^17^^,^^18^ enable volumetric analysis of melanin in vivo*.* Our group further investigated spectroscopic analysis of low melanin concentrations using visible light OCT and Mie scattering theory.^19^ Each of these volumetric imaging techniques, however, rely on more sophisticated technology not yet widely used in clinical practice. To address this point, we developed two new OCT biomarkers, a feature of interest based on traditional OCT intensity signals and a novel spectroscopic OCT metric for tracking chromophore concentration longitudinally. Although the intensity feature can only be observed in the eye, the spectroscopic metric was first validated in a phantom before applying it in vivo. These biomarkers were designed to be compatible with current clinical OCT devices for future clinical translation.

Here we examine longitudinal changes in the RPE and choroid of the superoxide dismutase 1 knockout (SOD1^−/−^) mouse model using our new intensity and spectroscopic OCT biomarkers and compare them to PS-OCT as a gold standard. The SOD1^−/−^ model is susceptible to elevated oxidative stress due to the knockout of SOD1 antioxidant enzymes and has been used as a model for AMD.^20^^,^^21^ In our previous examinations of this model, we found differences in retinal thickness and the formation of drusen-like lesions in the inner retina^22^; however, we focus this time on choroidal changes, with particular attention to melanin content, which may provide new insights into the mechanisms related to dry AMD. By characterizing the longitudinal changes in both new and existing OCT metrics related to melanin content in the SOD1 knockout mouse model, we demonstrate new clinical value for studying AMD using OCT.

## Methods and Materials

### Experimental Procedures

#### Phantom Imaging

**Figure 1.**
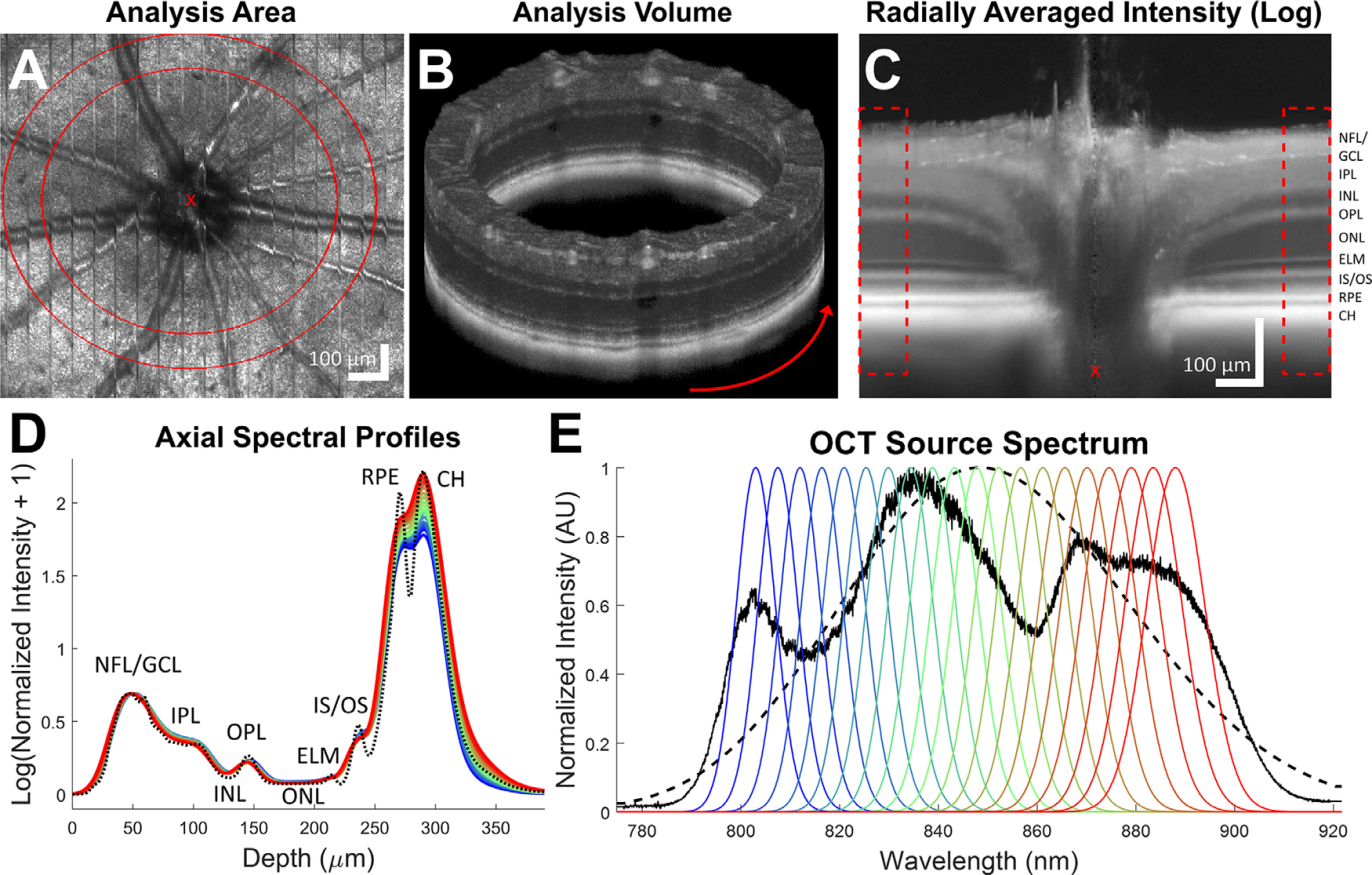
Processing pipeline for transverse averaging. A-lines are averaged within a hollow cylinder centered on the optic nerve head (*red X*) as denoted by the *red lines* in the en-face mean intensity projection (**A**) and shown in three dimensions (**B**). The radially averaged intensity cross-section (**C**), where the x axis denotes radial distance from the optic nerve head (*red X*), shows the specific ROI (*red dashed lines*) used for the resulting averaged and normalized axial profiles (**D**). Standard intensity (*dashed black lines*) and 20 spectral profiles (*colored lines*) from 803 nm (*blue*) to 888 nm (*red*) were generated from the Gaussian spectral windows shown in **E**. NFL, nerve fiber layer; GCL, ganglion cell layer; IPL, inner plexiform layer; INL, inner nuclear layer; OPL, outer plexiform layer; ONL, outer nuclear layer; ELM, external limiting membrane; IS/OS, inner segment/outer segment; CH, choroid.

**Figure 2.**
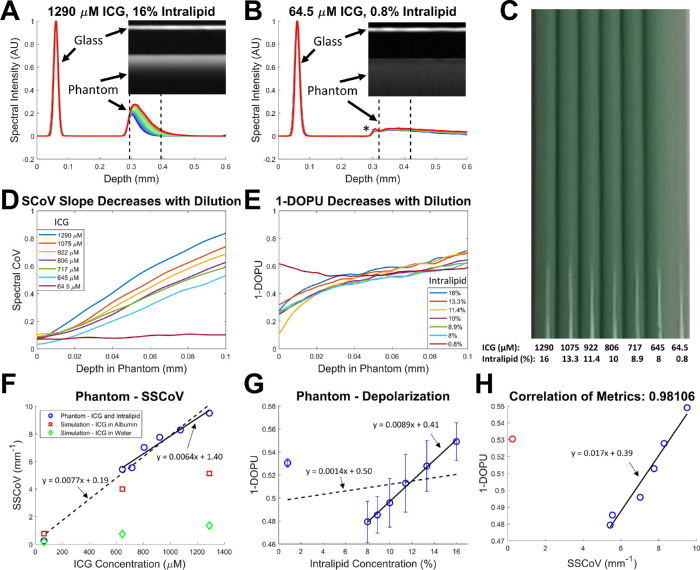
Spectroscopic and polarization-sensitive OCT imaging of Intralipid and indocyanine green (ICG) phantoms show correlation between spectral and depolarization metrics. Intensity depth profiles of individual spectral channels are normalized to the initial glass reflection and shown for the initial phantom mixture (**A**) and a 1:19 dilution with saline solution (**B**). Lines are coded from *blue* to *red* for shorter to longer wavelength bands and the asterisk in B identifies a small reflection from the glass-phantom interface. (**C**) A white light photo of the phantom dilutions sampled into glass capillary tubes. SCoV (**D**) and 1-DOPU metrics (**E**) are shown for 100 µm below the phantom surface marked by the dashed lines in A and B. SSCoV (**F**) and average 1-DOPU values (**G**) are then plotted against ICG concentration and Intralipid concentration, respectively. SSCoV values obtained from simulations of ICG in albumin and ICG in water are plotted as *red squares* and *green diamonds*, respectively. Depolarization standard deviations across the averaged depth range are shown as *error bars*. (**H**) A correlation plot of 1-DOPU and SSCoV metrics shows strong linear correlation using the Pearson correlation coefficient when excluding the most diluted sample. Linear least squares fits are plotted for all data points (*dashed line*) and excluding the most diluted sample (*solid line*).

A set of phantoms was produced for validating the new spectral metrics described below. These phantoms used serial dilution of a mixture of indocyanine green (ICG) dye and Intralipid 20% with saline solution. ICG is a common fluorescent dye used in ophthalmic imaging, which was used here to provide a variable absorption profile across the measured wavelength band from 803 nm to 888 nm (Fig. 1E). Intralipid is an emulsion of fat particles that was used here to backscatter and depolarize^23^ the incident light. Intralipid does not have strong spectral characteristics and is not expected to interfere with the ICG spectral signal. A mixture of 5 mg/mL ICG and Intralipid 20% was initially prepared at a 1:4 ratio and diluted by saline solution in ratios of 1:4:1, 1:4:2, 1:4:3, 1:4:4, 1:4:5, and 1:4:95, yielding concentrations of 1290 µM, 1075 µM, 922 µM, 806 µM, 717 µM, 645 µM, and 64.5 µM for ICG and 16%, 13.3%, 11.4%, 10%, 8.9%, 8%, and 0.8% for Intralipid (Fig. 2C). Each dilution was freshly mixed, collected in a glass capillary tube, and imaged in a fixed mount using a custom-built PS-OCT system.^24^ The combination of Intralipid and ICG in a single phantom enables both depolarization and spectral characteristics to be measured simultaneously in a single scan. This ensures that the dilution of both components is identical and that other sources of variance such as phantom orientation are reduced. Simulations of expected spectral metrics were also performed based on known ICG absorption profiles in albumin and water at 1290 µM, 645 µM, and 64.5 µM concentrations.^25^ These simulations used the Beer-Lambert law with the known absorption coefficients at 20 wavelengths corresponding to the full bandwidth of our OCT system to estimate the spectral intensities up to 200 µm in depth within these different solutions. Because OCT measures backscattered light, the roundtrip distance into and out of the medium is used in this simulation.

#### Animal Imaging

This study further investigates the raw data produced by a previously reported study on longitudinal changes in SOD1 knockout mice in the inner retina.^22^ As such, the experimental procedures can be found elsewhere. In short, SOD1 knockout and wildtype mice were imaged noninvasively and longitudinally under isoflurane anesthesia using our rodent PS-OCT ophthalmoscope.^24^ For this study, a subset of 8 mice (4 SOD1^−/−^ and 4 SOD1^+/+^ wildtype littermates, ages 5 to 17 months) were selected for further analysis. These mice were chosen for having the highest number of longitudinal time points and OCT volumes across both eyes (23 SOD1^−/−^ volumes and 22 wildtype volumes). All experimental procedures have been approved by the ethics committee of the Medical University of Vienna and the Austrian Ministry of Education, Science, and Research (BMBWF/66.009/0272-V/3b/2019) and adhere to the ARVO Statement for the Use of Animals in Ophthalmic and Vision Research. By further examining existing data, we support the best practices of animal research (replacement, reduction, and refinement).

#### Histological Preparation

At the terminal endpoint of this study, tissue samples were acquired from 4 of the mice (2 SOD1^−/−^ and 2 wildtype, aged 71–75 weeks) directly after euthanasia by cervical dislocation. Eyes were enucleated, cut open, and immersed in 4% paraformaldehyde (PFA)/phosphate-buffered saline solution for 24 hours. After 4% paraformaldehyde in phosphate-buffered saline solution, they were dehydrated in graded ethanols and embedded in paraffin. Sections (3 µm thick) were cut along a dorsoventral meridian in three series, approximately 300 µm nasal to, 300 µm caudal to, or through the optic nerve head and stained with hematoxylin and eosin or, to highlight collagen, with Heidenhain's Azan trichrome stain. Micrographs from locations corresponding to approximately 300 µm dorsal eccentricity were acquired on a Zeiss Imager Z2 (Zeiss, Oberkochen, Germany) with an Axiocam MRm or MRc5 at identical exposure times.

### Data Processing

#### OCT Data Processing

OCT data were processed using standard methods including automated dispersion compensation,^26^ Gaussian spectral shaping,^27^ and motion compensation with flattening of the retina.^28^

#### Region of Interest (ROI) Selection and Reduction of Dimensionality

Noise reduction is a critical part of OCT data analysis due to the characteristic speckle patterns that are inherent to OCT data. The effects of speckle in OCT data can be reduced by averaging longitudinally or temporally, which is particularly important for spectral analysis, which typically requires a higher number of averages to produce reliable results.^29^^–^^31^ To obtain high-quality depth information, the dimensionality of the OCT data was reduced from three dimensions to one dimension. Volumes were reduced to depth profiles by averaging over all lateral positions within an annular ROI (Figs. 1A, 1B) centered on the optic nerve head (ONH) of each animal similar to our previous work.^32^ Here, the lateral coordinates of the center of the ONH were manually annotated in each volume, and a 90 µm thick ROI with an inner radius of 370 µm and an outer radius of 460 µm was used. This ROI was chosen after observing that the laminar retinal structure for these mice was more uniformly flat as a function of radius in this region (Fig. 1C). Averaging in this region ensures that the resulting depth profiles consistently retain sharp features (Fig. 1D). Traditional intensity, polarization-sensitive, and spectral depth profiles were obtained and investigated for progressive changes in the transgenic and control groups.

#### Intensity Processing and the RPE-Choroid Ratio

Intensity data were obtained using traditional processing methods applied to only the co-polarized OCT channel, which is comparable to what would be observed in a traditional OCT system. For visualization purposes, intensity images were generated using either lateral or radial averaging and are shown in log scale. Radial averaging was performed by reslicing the volumetric data in 0.5° increments laterally around the ONH, which served as the pivot point (red X in Figs. 1A, C). The new radial image stack was then averaged to show the laminar structure of the retina as a function of distance from the ONH. Intensity in the depth profiles was normalized by subtracting the background noise in the vitreous and dividing by the maximum signal in the nerve fiber layer and ganglion cell layers (NFL/GCL). In the phantom experiment, the intensity was normalized by subtracting the background noise in air and dividing by the maximum signal from the air-glass interface. Mean profiles across animals were computed after aligning all profiles using a cross-correlation algorithm. The relationship between RPE and choroidal intensity signals was measured as the maximum of the linear-scale RPE intensity divided by the maximum of the linear-scale choroidal intensity and is referred to here as the RC ratio.

#### Polarization Processing

Polarization processing used both the co- and cross-polarized OCT channels to obtain degree of polarization uniformity (DOPU) information,^33^ where a DOPU of 1 represents tissue that perfectly maintains the polarization of the light traveling through it, and a DOPU of 0 represents tissue that fully depolarizes the light as it passes through. DOPU calculations followed established formulae with noise bias correction^34^ and used a uniform smoothing kernel with a size of 4 × 4 × 4 pixels (z, x, y) or 8 × 8 × 10 µm. Mean DOPU profiles across animals were computed after shifting each profile by the same offsets used to align intensity profiles. To more easily present and correlate the DOPU information with the following spectral metrics, the metric 1-DOPU is used from here on as a measure of depolarization.^35^

#### Spectral Processing and the Slope of the Spectral Coefficient of Variation

Spectral processing methods used only the co-polarized OCT channel, similar to the intensity processing, to demonstrate that they are feasible using traditional OCT hardware. Spectral information was obtained by using a series of 20 narrow Gaussian windows in k-space^19^ with six times lower axial resolution than the standard intensity information, which has an axial resolution of 3.8 µm in tissue.^24^ These Gaussian windows were evenly spaced in wavelength with widths that were uniform in k-space. The spectral windows had an overlap of 57% to 71% by area in wavelength space. These parameters were selected to balance spectral specificity while retaining reasonable axial resolution for visualizing the retinal structure. Spectral profiles were background subtracted and normalized following the same procedure as the standard intensity profiles. Spectral profiles within each animal were then aligned using a cross-correlation algorithm applied to the superficial portion of the retinal spectral profiles to remove residual first order chromatic dispersion. Finally, spectral profiles for each animal were realigned to the standard intensity profile to ensure consistency of features across metrics.

Using these spectral profiles, a new metric that we call the slope of the spectral coefficient of variation (SSCoV) was derived. As the name suggests, the SSCoV is calculated by measuring the local slope of the coefficient of variation (CoV) profile across spectral channels, where the CoV is given as the standard deviation divided by the mean across linear-scale spectral intensities for each axial position (z):

$$\begin{array}{c} CoV\left(z\right)=\frac{\sigma_{\lambda }\left(z\right)}{\bar{I_{\lambda }}\left(z\right)}. \end{array}$$

The CoV describes the variability across spectral profiles similar to the standard deviation, however, normalizing by the mean spectral intensity makes the CoV unitless. This makes the CoV less dependent on the backscattered intensity and more reliably comparable across animals and time points. The spectral CoV (SCoV) increases as a function of depth when light travels through tissue that has a spectrally dependent absorption profile. Provided the tissue remains relatively homogenous over depth, the resulting axial slope of this spectral CoV profile can be used as a quantitative indicator of chromophore concentration. For the one dimensional SSCoV profiles and two dimensional SSCoV images in this work, a sliding axial window of 42 µm (21 pixels) was used for fitting the linear slope of the SCoV as a function of depth in the retina using a least squares method. The value of the resulting fit within the window was then assigned to the pixel centered in the axial window; hence, the odd number of pixels used. This window was chosen to suppress high frequency noise in the fits, particularly at the edges of different retinal layers where large intensity shifts occur, while minimizing the blurring of real spectral features. SSCoV was also compared across different animals longitudinally. For this analysis, the SSCoV was calculated over a fixed region of the choroid with a depth range of 22 µm (11 pixels), where the SSCoV signal was most stable. The choroid is optimal for this comparison because it is expected to have a higher chromophore concentration with a more homogenous distribution over a larger depth range in mice compared to the relatively thin RPE.^19^ These choroidal features should yield better SSCoV fits and more reliable data points compared to the RPE in the presence of longitudinal data quality fluctuations.

#### Cross-Sectional Image Processing

OCT cross-sectional images were averaged over 50 B-scans (125 µm), with five repeats for each B-scan, and normalized to the maximum signal at the retinal surface in the same manner described above. Depolarization and SSCoV images were further intensity compensated below a given intensity threshold to reduce the effects of noise for visualization purposes. This threshold was set at 0.2 normalized intensity units in linear scale to balance removal of noise while preserving real signal.

#### Statistics

Statistical analysis was performed on intensity, depolarization, and spectral metrics both independent of and dependent on mouse age. For age-independent analysis the Wilcoxon rank sum test was used to evaluate the distributions of metrics between groups. For age-dependent analysis, least squares linear fits and ranges for the 95% confidence intervals were calculated for each group. The *t*-statistics of the fits were used to test whether slopes were significantly age-dependent, and the *t*-statistics of the differences between the control and SOD1**^−^**^/^**^−^** fits were used to determine whether slopes were significantly different from each other. For all tests, a *P* value of less than 0.05 was considered statistically significant. Correlations of metrics against each other are reported using the Pearson correlation coefficient.

## Results

In this study, a new spectroscopic OCT metric was validated in a phantom and applied alongside a new intensity feature and established polarization metrics to study changes in the RPE and choroid of the SOD1 mouse model longitudinally. Histological analysis was performed to support in vivo findings.

### Phantom Validation

An ICG-Intralipid phantom was used to characterize the performance of the new SSCoV metric and compare it against PS-OCT metrics that have been proved capable of identifying retinal melanin.^14^^,^^15^ At the full concentration of the phantom, spectral divergence due to absorption is clearly visible (Fig. 2A), but this becomes less pronounced at lower concentrations because of less absorption (Fig. 2B). The spectral CoV and 1-DOPU depolarization metric both increase as a function of depth within the phantom (Figs. 2D, 2E). SSCoV increases with ICG concentration in an approximately linear fashion with a slight difference observed between linear fits that include or exclude the lowest concentration data point (Fig. 2F). This lowest ICG concentration point has the highest concentration of saline solution and agrees more closely with the simulation of ICG in water compared to the other concentrations. Depolarization also increases with Intralipid concentration with the exception of the lowest concentration data point (Fig. 2G), resulting in a strong correlation of 0.98 between SSCoV and depolarization at higher concentrations of the phantom (Fig. 2H).

### SOD1 Findings

Intensity, polarization, and spectral analysis of SOD1**^−^**^/^**^−^** and control OCT data yielded several statistically significant findings.

#### Cross-Sectional Images

**Figure 3.**
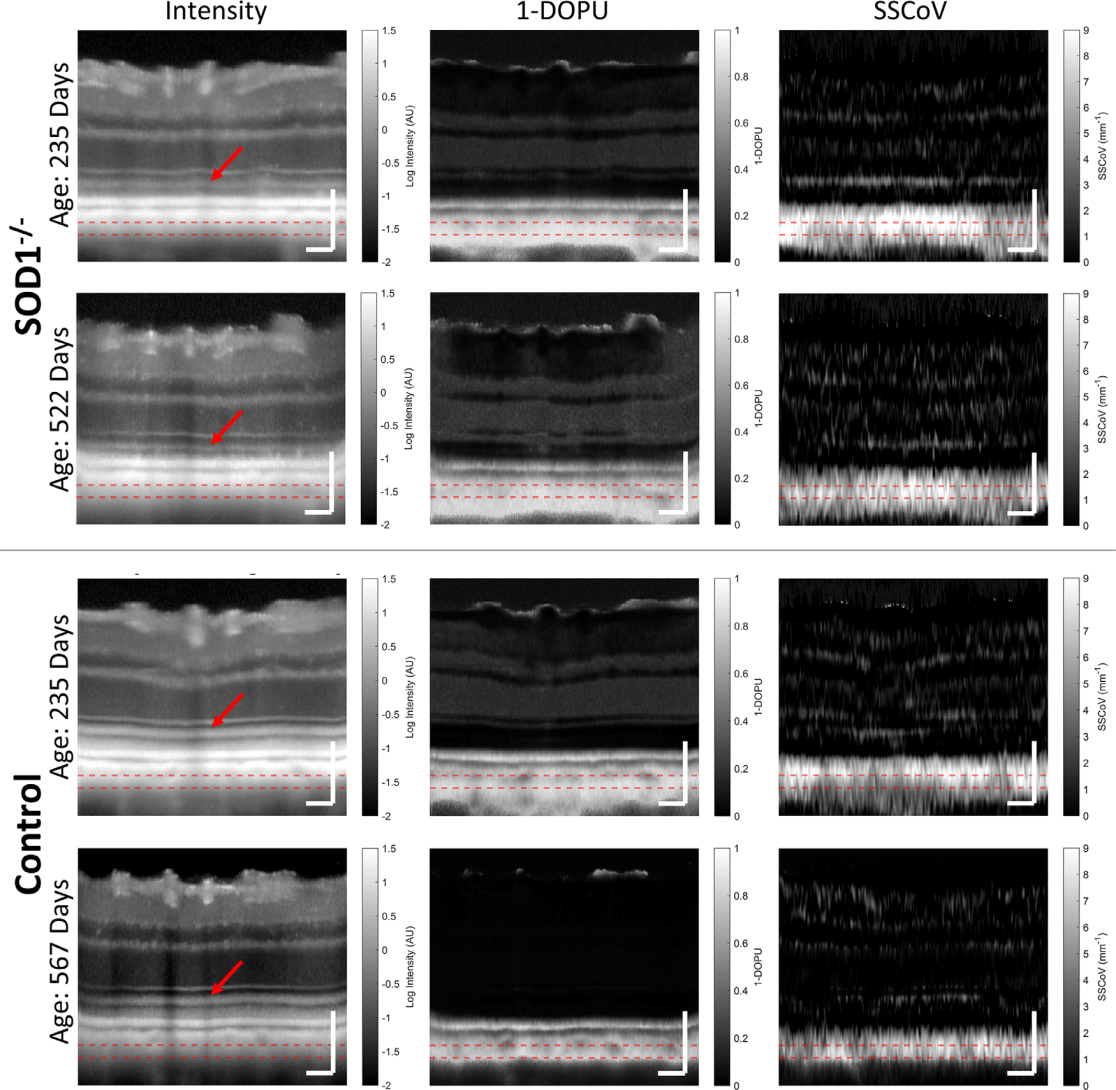
Intensity, depolarization, and SSCoV images from SOD1^−/−^ and control mouse eyes at similar positions and at two time points at the beginning and end of the longitudinal study. *Red arrows* point to the inner and outer photoreceptor segment junction, and *red dashed lines* mark the region of interest further examined in Figure 4 and Figure 5. *Scale bar*: 100 µm.

Cross-sectional images of each analysis method in this study were generated at similar locations and time points for control and knockout mice. In the intensity cross-sections, it was observed that the inner and outer segments were substantially brighter in the control mouse than the knockout mouse (Fig. 3, red arrows). Additionally, flow voids in the vicinity of the choroidal region of interest (Fig. 3, red lines), corresponding to large choroidal vessels, appear more visible in these control cross-sections. The RPE and choroidal 1-DOPU signals are clearly distinguishable from each other and have substantially higher signal than in the inner retina. Low signal regions corresponding to large vessels in the choroid were also resolvable using 1-DOPU. The RPE and choroidal SSCoV signals are similarly higher than the inner retinal signals, but they are not clearly distinguishable from each other. Flow voids also appear to affect SSCoV images, but individual vessels are less defined.

#### Depth Profiles

**Figure 4.**
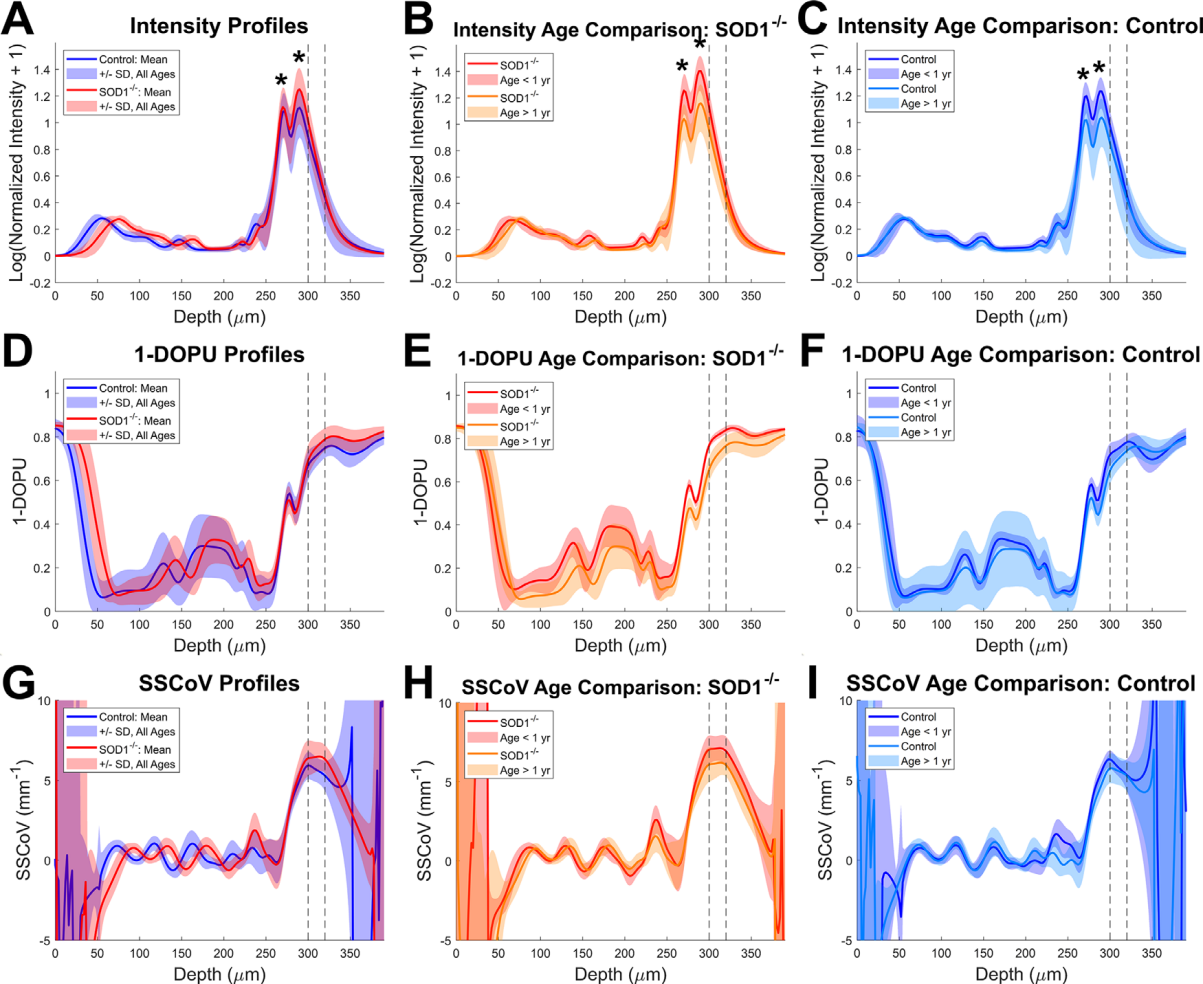
Mean axial profiles across all ages and eyes for control (*blue*) and transgenic (*red*) mice for intensity (**A**), 1-DOPU (**D**), and the slope of the spectral coefficient of variation (SSCoV) (**G**). Data were further sorted into age groups of less than 1 year or greater than 1 year for SOD1**^−^**^/^**^−^** animals (**B**, **E**, **H**) and control animals (**C**, **F**, **I**). Retinal and choroidal maximum intensities are marked by *asterisks*. *Dashed black lines* correlate to the depth of the *dashed red lines* in Figure 3 and the region of interest further investigated in Figure 5.

In the depth profiles of an individual data set, spectral intensity and traditional intensity signals are in good agreement within the inner retina despite the lower resolution of spectral signals (Fig. 1D). The spectral signals themselves are consistent within the inner retina but diverge in the RPE and choroid. Average depth profiles of each metric were also generated for control and knockout mice using data from all the mice in this study and were further broken into age groups of less than and greater than one year (Fig. 4). Intensity profiles of the two animal groups revealed clear retinal thinning within the SOD1**^−^**^/^**^−^** mice (Fig. 4A). This thinning appears to continue over time within the disease group (Fig. 4B), but not the control group (Fig. 4C). Nevertheless, good agreement between disease and control groups are observed with the exception of choroidal peak intensity, which was notably higher in the disease group. Depolarization, measured as 1-DOPU, was low within the inner retina and high within and below the RPE, where melanin is expected (Figs. 4D–F). The high depolarization values in the depth range from 0 to 50 µm occur in the vitreous and are due to noise. Similarly, SSCoV profiles in the depth range from 0 to 50 µm are initially erratic due to noise in the vitreous, but quickly settle and oscillate around 0 mm^−1^ within the inner retina (Figs. 4G–I). SSCoV increases starting at the RPE and eventually plateaus in the choroidal and scleral regions.

#### Longitudinal Changes and Correlation of Metrics

**Figure 5.**
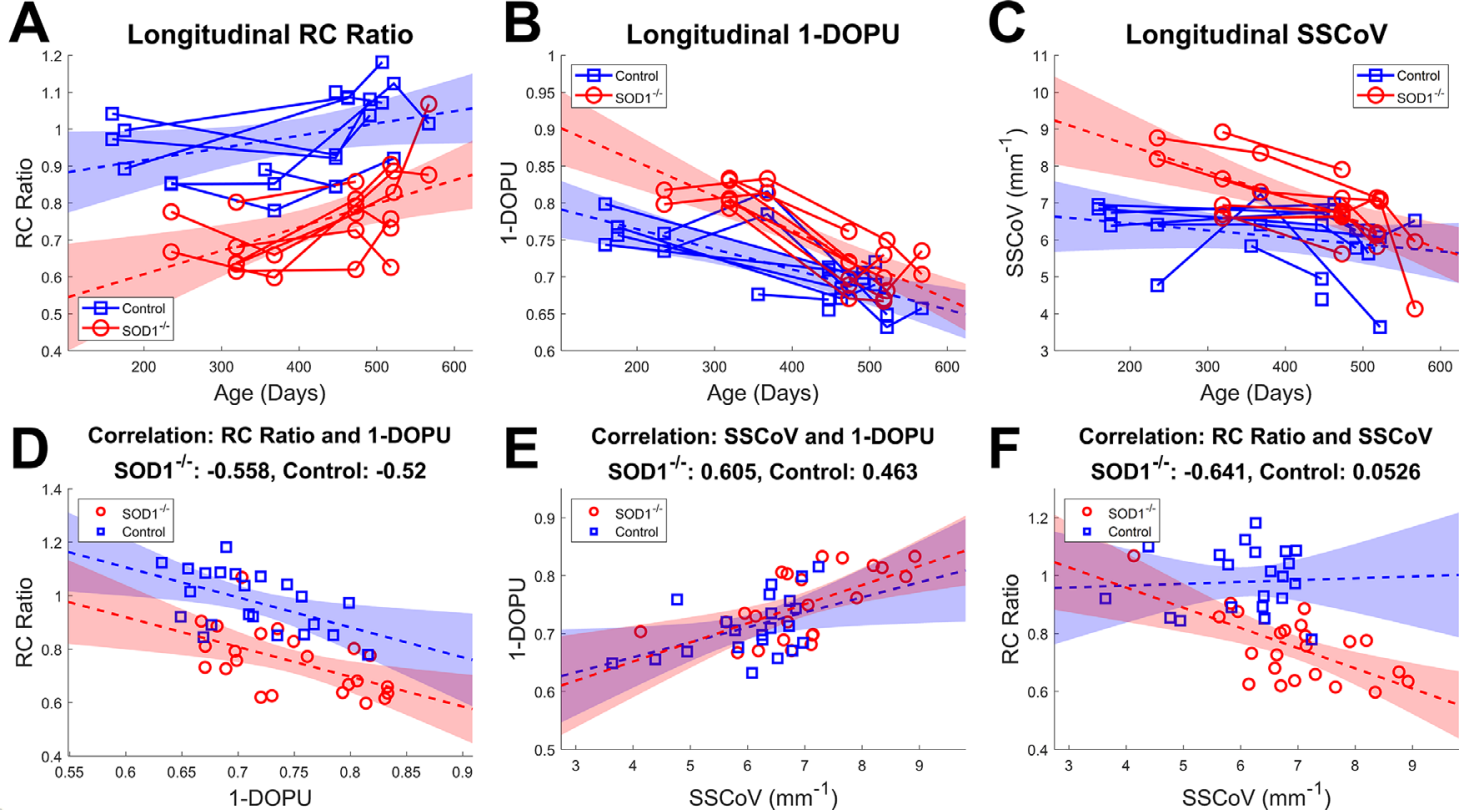
Longitudinal trends of the RC ratio (**A**), depolarization (**B**), and SSCoV (**C**) as a function of age. Each *solid line* contains the longitudinal data points from one of the eyes of the studied mice. Correlation plots comparing each of the metrics against each other for all data points are also shown (**D**–**F**). *Dotted lines* and colored regions show the linear least squares fit and 95% confidence interval, respectively, for each experimental animal group.

Longitudinal changes in the choroidal region of interest (dashed lines in Fig. 3 and Fig. 4) were further evaluated for each metric (Figs. 5A–C and Table 1). RC ratio increased with age in the knockout group (*P* < 0.005), but did not show a significant age dependence in the control group (*P* = 0.06; Fig. 5A; Table 1). When comparing the rates of change of the RC ratio for disease and control groups against each other directly, no statistically significant difference was detected (*P* = 0.15); however, the RC ratio itself was significantly higher in the control group (*P* < 0.0005). Depolarization demonstrated a decrease with age in the marked ROI for both groups (*P* < 0.0005) with statistically different slopes (*P* < 0.05) where the knockout group decreased more rapidly than the control group (Fig. 5B, Table 1). The difference between groups independent of age was also found to be significant (*P* < 0.05) with higher 1-DOPU values associated with the knockout mice. SSCoV decreased in the knockout group with age (*P* < 0.0005), but age-dependent trends were not significant for the control group (*P* = 0.21; Fig. 5C, Table 1). SSCoV declined significantly more rapidly with age in the knockout group compared to the control group (*P* < 0.05, Table 1). Similar to 1-DOPU, SSCoV values were also significantly higher in the knockout group overall compared to the control group (*P* < 0.05).

**Table 1.** Measured Values and Statistical Significances for Figures 5A–C

	Mean ± (Standard Deviation)		*P* Value	Slope ± (95% Confidence Interval) in Units Per Year		*P* Value		
	Control	SOD1**^−^**^/^**^−^**	Between Groups	Control	SOD1**^−^**^/^**^−^**	Control	SOD1**^−^**^/^**^−^**	Between Groups
RC Ratio	0.985 (0.089)	0.752 (0.065)	**0.000155**	0.121 (0.127)	0.232 (0.154)	0.0604	**0.00499**	0.148
1-DOPU	0.707 (0.030)	0.747 (0.36)	**0.0207**	−0.099 (0.045)	−0.170 (0.053)	**0.000155**	**1.36 × 10^−^^6^**	**0.0117**
SSCoV (mm^−1^)	5.91 (0.81)	6.95 (0.73)	**0.0104**	−0.69 (1.10)	−2.56 (1.26)	0.206	**0.000397**	**0.00586**

Statistically significant *P* values (*P* < 0.05) have been bolded.

Analysis of the correlation between metrics revealed several additional relationships (Figs. 5D–F, Table 2). First, the SOD1**^−^**^/^**^−^** and control mice both demonstrated significant negative correlations (−0.56 and −0.52 respectively, *P* < 0.05) between RC ratio and 1-DOPU with similar slopes but different offsets (Fig. 5D). Second, significant positive correlations were observed in both SOD1**^−^**^/^**^−^** and control mice between SSCoV and 1-DOPU metrics (0.61 and 0.45, respectively; *P* < 0.05), and this correlation was not statistically different between animal groups (*P* = 0.46; Fig. 5E). Finally, the correlation between RC ratio and SSCoV demonstrated significantly different characteristics for the two animal groups (*P* < 0.05). The controls demonstrated no correlation between these metrics (0.053; *P* = 0.82), but the SOD1**^−^**^/^**^−^** group showed a strong negative correlation (−0.64; *P* < 0.005; Fig. 5F).

**Table 2.** Measured Pearson Correlation Coefficients and Statistical Significances for Figures 5D–F

	Correlation Coefficient		Slope ± (95% Confidence Interval)		*P* Value		
	Control	SOD1**^−^**^/^**^−^**	Control	SOD1**^−^**^/^**^−^**	Control	SOD1^−/−^	Between Groups
							
RC Ratio vs 1-DOPU	−0.520	−0.558	−1.12 (0.86)	−1.11 (0.75)	**0.0132**	**0.00567**	**2.05 × 10^−^^31^**
1-DOPU vs SSCoV (mm^−1^)	0.463	0.605	0.0259 (0.0231)	0.0330 (0.0197)	**0.0302**	**0.00223**	0.461
RC Ratio vs SSCoV (mm^−1^)	0.053	−0.641	0.0064 (0.0562)	−0.0697 (0.0379)	0.816	**0.000985**	**0.0105**

Statistically significant *P* values (*P* < 0.05) have been bolded.

#### Histology

**Figure 6.**
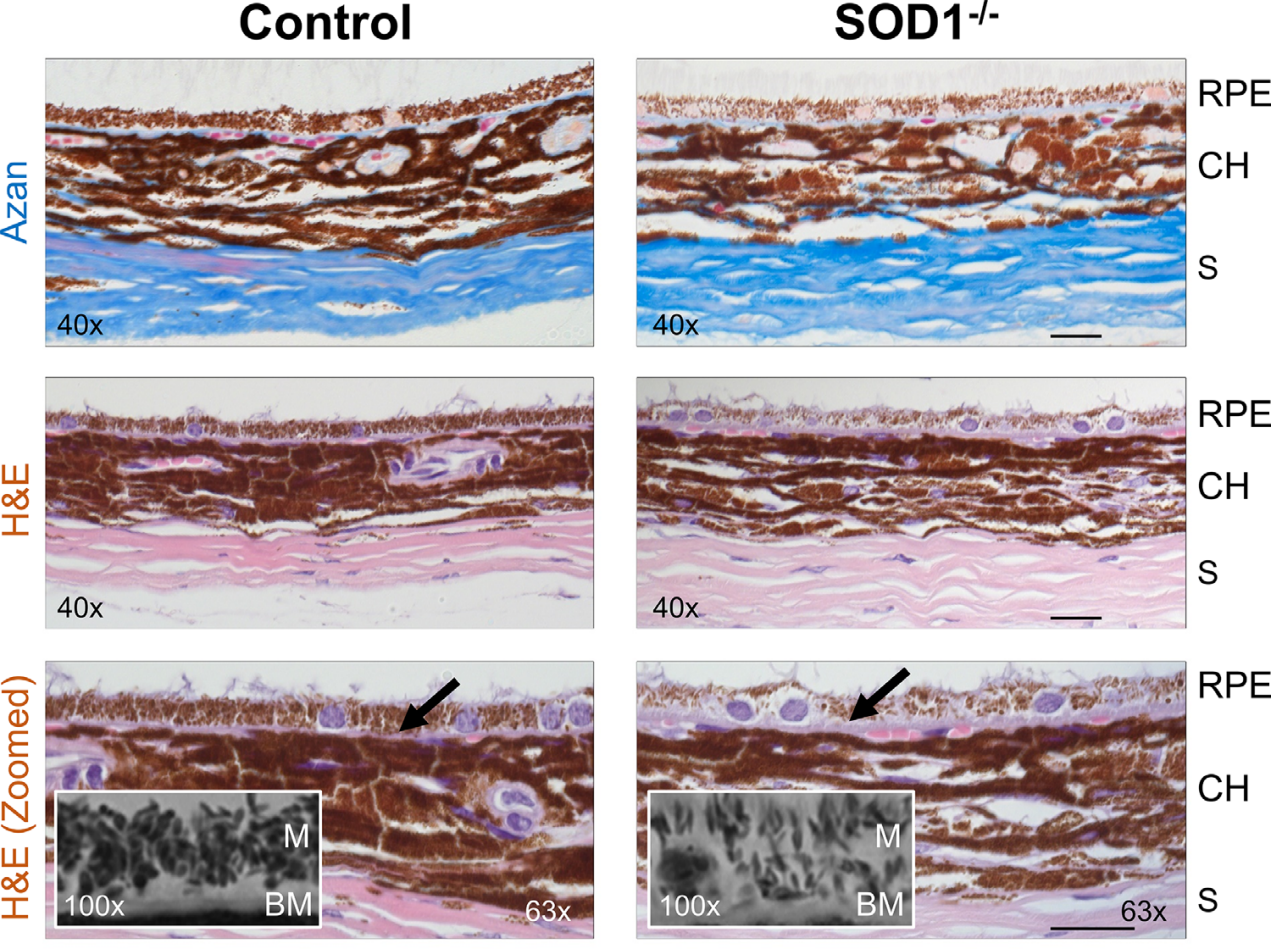
Histology of the eyes shows melanin content in the RPE and choroid (CH) of age-matched SOD1**^−^**^/^**^−^** and control mice. Sections of each animal were stained with Azan trichrome to highlight collagen in the sclera (*S*) and Bruch's membrane (*BM*), or with hematoxylin and eosin (*H&E*). Additional higher magnification scans of the slices shown in the third row were taken to highlight the thickness of the Bruch's membrane (*black arrows*) and the distribution of melanin granules (*M*) in the RPE. Width of insets and scale bars: 20 µm.

Histological analysis demonstrated a marked decrease in RPE melanin content in the SOD1**^−^**^/^**^−^** mice compared to controls (Fig. 6). RPE melanin size and shape distribution also appeared to differ in the SOD1**^−^**^/^**^−^** group with a higher prevalence of longer and narrower granules compared to rounder granules found in controls observed in the Figure 6 insets. Compared to differences in the RPE, Choroidal melanin distributions appeared to be more similar from visual examination. Thickening of Bruch's membrane (Fig. 6, arrowheads) was also noted in the disease mice.

## Discussion

The trends and correlations observed using intensity, depolarization, and spectral analysis methods are each correlated to expected and observed changes in retinal melanin content. Here we interpret and contextualize the findings beginning with the phantom validation of SSCoV before discussing findings from the longitudinal study of the SOD1 knockout model.

### Phantom Findings

The results of the phantom experiment demonstrated that for a solution that both depolarizes and has a spectrally variant absorption profile, both SSCoV and 1-DOPU can be used to non-invasively probe concentration with linear response curves and strong correlation between the two metrics within a certain dynamic range. At the lowest concentration, the phantom produced an abnormally high 1-DOPU measurement, which may be due to low signal. When dominated by noise, the 1-DOPU signal should trend upwards. Furthermore, reflections from the glass-phantom interface become visible at low phantom concentrations, as shown as an extra peak in the intensity profile (Fig. 2B, asterisk) and may potentially disrupt polarization measurements. Of interest, the SSCoV phantom measurements were generally higher than simulated values for ICG in albumin and in water (Fig. 2F). This is due to the strong solvent dependence of ICG on the absorption profile^25^^,^^36^ and indicates a larger wavelength dependence or stronger absorption in a lipid solution. This finding is supported in part by previous literature which demonstrates higher fluorescence yield from lipid-bound ICG^37^; however, no absorption profile could be found. The lowest concentration data point is in close agreement with the simulation of ICG in water because at the lowest concentration, the phantom is 95% saline solution, which likely shifted the absorption profile back towards the simulated shape and magnitude. It is important to note that the SSCoV does not strictly increase linearly with depth but is in fact curved over a large enough depth range or with a strong enough absorber. Nevertheless, for the concentrations used in the phantom, which reflect a similar range of SSCoV values found in the animal experiments, a linear estimation is fair over a meaningful depth range. While not the same chromophore studied in vivo, ICG was chosen for this phantom because of its known absorption profile, high homogeneity in a phantom, and to demonstrate that SSCoV is nonspecific to a particular chromophore.

### SOD1 Knockout Findings

#### Intensity

It is clear from the comparison of control and SOD1 knockout axial profiles (Figs. 4A–C) that there are substantial differences in retinal thickness, which agree well with our previous findings on these same mice.^22^ Retinal thinning combined with the lower reflectivity of the inner and outer segments for the mouse shown in Figure 3 indicate damage to the photoreceptors of SOD1 knockout mice.^38^ There are also apparent decreases in the RPE and choroidal intensities over time for both control and disease mouse groups (Figs. 4A–C). The mechanism by which this reflectivity changes over time is not clear due to combined absorption and scattering effects, but may be related to loss of scattering power due to the age-related degeneration of melanin in these layers.^4^ It is also possible that the normalization method, which divides the axial signal by its maximum intensity within the superficial retina, could cause a decrease in signal within the RPE and choroid if inner retinal signals increased over time.

To further investigate RPE and choroidal intensity effects, the RC ratio was used to mitigate age-dependent drift in intensity caused by variations in experimental conditions such as beam alignment, cataract formation, and image quality, rather than the underlying retinal tissue. RC ratio was found to be an appealing metric due to its ease of measurement and inherently normalized values. This normalization enables robust comparison across animals and over time despite intensity drift, which decreased in some animals by as much as ∼3.5 times in the RPE and choroid despite normalizing to the retinal surface. RC ratio on the other hand varies only as much as ∼1.5 times within each eye over the course of the study and clearly differentiates the knockout and control groups. In addition to RPE and choroidal reflectivity, we believe the RC ratio may be most strongly associated with the melanin content of the RPE. Because the RPE is a thin layer, RPE melanin related absorption will not strongly affect the RPE intensity peak. Similarly, the peak choroidal signals are expected to be located near the choroidal surface due to absorption effects at deeper regions of the choroid, so the effects of choroidal melanin absorption on the choroidal peak are expected to be minimal. The choroidal peak would however be affected by the absorption of the incoming light by the melanin in the RPE above. Under these assumptions, the dominant effect on RC ratio would come from RPE melanin where higher RPE melanin content has a compound effect leading to higher RPE backscattering and less light propagating to the choroid, both of which result in a higher RC ratio. The observation that RC ratio was lower in the SOD1 knockout group (Fig. 5) supports this interpretation. It has been established in previous literature that one of the hallmarks of the SOD1 mouse model is a progressive degeneration of the RPE because of increased oxidative stress,^20^^,^^21^ which accelerates oxidative degradation of melanin.^39^ The degeneration of RPE melanin was clear from our own histological analysis (Fig. 6). Interestingly, the RC ratio changed significantly with age in the knockout group, but not for the controls. Although the increase in RC ratio with age could under other circumstances suggest an increase in melanin density over time, this is not realistic given the established degeneration of melanin with age^4^ and our histological results (Fig. 6). Instead, it is most likely due to uneven decreases in RPE and choroidal intensity signals with age, where more rapid choroidal signal loss would lead to increased RC ratio over time. Regardless of the exact cause, the RC ratio was clearly capable of separating the two experimental groups and therefore may be of value for future study as an AMD biomarker.

#### Depolarization and SSCoV

Polarization and spectral analysis of the choroid demonstrated similar longitudinal trends between both metrics: 1-DOPU and SSCoV were both initially higher and both decreased over time more quickly in the SOD1 knockout group compared to controls (Fig. 5). As proxies for melanin content, the more rapid decreases in 1-DOPU and SSCoV signals are supported by previous literature, which notes that melanin degeneration is more severe in an environment with higher levels of oxidative stress.^39^ Because SOD1 is an important enzyme for reducing oxidative stress,^40^ it is expected that the SOD1 knockout mice would experience more rapid melanin degeneration compared to controls. The difference in severity of RPE melanin degradation by the endpoint of the study is clear from the histological images (Fig. 6), which show lower melanin density in the SOD1 knockout mice compared to controls. It is important to note that the potential degradation of the OCT data quality and increase of noise with age, primarily due to age-dependent effects such as cataract formation, would actually have the opposite effect on the depolarization trends in severe cases, making 1-DOPU appear higher within the choroid with time. This means that while optical quality may still play a role, the observed trends are strong enough to counteract the potential influence of reduced data quality in this study.

While the finding of more rapid melanin degeneration in SOD1 knockout mice is supported by the literature, the higher initial 1-DOPU and SSCoV values in the SOD1 mice, are somewhat paradoxical. Higher values for these metrics would appear to indicate higher rather than lower melanin content in the SOD1 knockout mice at first glance, which is not reasonable given that RPE degeneration is expected around the earliest time points in our study.^21^ Although the source of this discrepancy is not yet clear, we have identified two possible effects that may cause or at least influence this finding. The first is the apparent change in shape distribution for the melanin granules that was observed in the RPE (Fig. 6). It is known that particle size, shape, and orientation play an important role in depolarizing,^15^^,^^41^ absorbing,^42^ and scattering incoming light with wavelength-dependent scattering profiles determined by Mie theory.^19^ In a simulation of spherical and ellipsoidal melanosomes with randomly distributed orientations, it was shown that the ellipsoidal melanosomes had an extinction coefficient that was approximately four times higher than their spherical counterparts for the wavelengths used in this study.^42^ This may directly increase the SSCoV signal despite a lower overall melanin concentration, provided that the SOD1 knockout mice have a higher concentration of ellipsoidal melanosomes. Changes in the polarization-dependent absorption profile may also play a role given that spectral analysis is applied to only the co-polarized OCT channel. If the change in melanin shape distribution resulted in a higher degree of depolarization and a stronger wavelength dependence in the intensity of the SOD1 knockout mice, this could explain the higher 1-DOPU and SSCoV signals despite the lower melanin density. The other possible explanation relates to the flow voids more commonly observed in the control cross-sections compared to the SOD1 cross-sections in Figure 3. It is possible that the SOD1 model demonstrates choroidal atrophy,^3^ which could account for the lower prevalence of flow voids, which have been linked to large choroidal vessels. These flow voids clearly decrease the 1-DOPU and SSCoV signals (Fig. 3). A lower density of choroidal vasculature could therefore result in higher average depolarization and SSCoV signals even with lower melanin density if the melanin-containing tissue makes up a larger volumetric portion of the choroid itself.

#### Correlation Analysis

The correlation between RC ratio, depolarization, and SSCoV metrics (Fig. 5) revealed some interesting trends which provide insights into the contrast mechanisms. First, there is a clear and strong correlation between depolarization and SSCoV which is statistically indistinguishable between the two groups (Fig. 5E). This supports the use of SSCoV as a measure for melanin concentration. There is also a strong correlation between depolarization and RC ratio with similar slopes but different offsets for the two groups. This may be due to some overlap in the origins of the two signals. As discussed above, the RC ratio is dependent on both RPE and choroidal intensities, however RPE melanin is expected to be the largest contributor to this signal. Conversely, depolarization measurements at this depth are expected to be primarily driven by depolarization from choroidal melanin, however depolarization by RPE melanin influences the signal as well. The influence of RPE melanin on depolarization may explain the steeper decreases in depolarization over time compared to SSCoV, which is a more localized measurement because of its self-normalizing nature, which makes SSCoV less influenced by the signals above it. This effect may also explain why there is a significant decrease in depolarization but no significant change in SSCoV in the control group due to normal changes in RPE melanin over time. The correlation between SSCoV and RC ratio in the SOD1**^−^**^/^**^−^** group but not in the controls is particularly interesting and may indicate that choroidal melanin degeneration plays a role in the RC ratio signals of the knockout mice, but not in controls. This supports the idea that the increasing RC ratio with age, which is only significant in the knockout mice, is caused by choroidal melanin degeneration.

### SSCoV Performance

SSCoV is used here to investigate melanin concentration in the choroid, however it is also broadly applicable to any chromophore with a variable absorption profile across the source bandwidth. One key aspect of the SSCoV metric is that it is nonspecific, which means it can be applied to identify any chromophores with spectrally dependent absorption or scattering profiles. Unlike other spectral analysis methods which use model-based fitting of the spectral signal,^29^^,^^30^ a priori assumptions of chromophore spectra, which can sometimes change depending on the local environment, are not required. This makes application of the SSCoV metric easy to fully automate and it can be applied to traditional OCT volumetric scans, as presented here. This metric does have the drawback of not being able to differentiate between multiple chromophores present within a given volume, but it can be used to guide further investigation of chromophores using other spectral analysis methods.

Although visible light OCT is often used for spectroscopy,^43^^–^^45^ traditional clinical OCT uses longer wavelength sources and does not typically make use of the spectroscopic information intrinsically provided by OCT. Our findings using our longer wavelength system at 840 nm suggest potential clinical value for spectroscopic analysis of pigmentation using near infrared light. While the axial resolution of the SSCoV images was lower than for depolarization and did not resolve a clear difference between the RPE and choroid (Fig. 3), this was influenced primarily by the large (42 µm) axial window used for fitting the slope of the SCoV signal and could potentially be improved with better averaging. Better averaging and/or higher axial resolution may also improve the SSCoV signal in the RPE which was sometimes less visible in cross-section at lower signal levels due to its thinness and the lower melanin concentration compared to the choroid as seen in histology (Fig. 6). The spectral windows selected for this study produced a high degree of spectral overlap (57% to 71%). The relatively high sampling density provided more spectral points for fitting and comes at no cost other than additional computation time and storage space. Future studies may better optimize the selection of spectral window parameters.

### Melanosome Quantification

One potentially interesting direction for future research is the quantification of melanosome loss using these methods. Quantification of melanosome loss using the SSCoV and 1-DOPU metrics is theoretically possible given that both have a known zero bound, which gives a point of reference for extrapolating melanosome loss over time. RC ratio, however, lacks this feature, making it poorly suited for this task. The real difficulty in establishing a true quantitative estimate of melanosome loss lies in the assumptions that must be made regarding the measured values and the underlying tissue structure. Although we demonstrated a clear relationship between 1-DOPU and SSCoV and the resulting phantom concentrations, this addresses a very controlled case and does not take into account biological effects such as melanosome loss of different sizes and shapes. It is also not yet known how linear these metrics will be with melanosome concentration because of potential saturation or shadowing effects at higher concentrations. To perform quantitative melanosome loss measurements using these techniques, a future study would be needed to investigate the effects of melanosome shape, orientation, and concentration on these signals.

## Conclusions

In this work we demonstrate a new spectral analysis method, SSCoV, as a nonspecific indicator of chromophore concentration in tissue. This metric was validated using simulations and phantoms and applied alongside traditional intensity and polarization imaging schemes to further investigate data from a longitudinal animal study. Our previous work demonstrated differences between the SOD1**^−^**^/^**^−^** mouse model and control mice in the inner retina. Here we demonstrated new findings including a statistically significant difference between intensity patterns in the RPE and choroid between control and SOD1**^−^**^/^**^−^** mice using the RC ratio. This new metric was robust against intensity drift and clearly differentiated the two mouse groups, potentially indicating consistently lower RPE melanin in the knockout mice. Analysis of choroidal chromophore content using both SSCoV and depolarization measurements demonstrated statistically significant differences, which may reflect choroidal chromophore degradation over time. These results were further supported by histological analysis which demonstrated decreases in melanin concentration and differences in RPE melanin granule shape in knockout mice. Because they were designed around the limitations of modern clinical OCT systems, the SSCoV metric and RC ratio feature may be valuable for extracting new biomarker information from clinical OCT data without needing to replace existing OCT hardware. The clinical potential of these methods on human OCT data will be evaluated in a future study.
